# TGF-β1 induced deficiency of linc00261 promotes epithelial–mesenchymal-transition and stemness of hepatocellular carcinoma via modulating SMAD3

**DOI:** 10.1186/s12967-022-03276-z

**Published:** 2022-02-05

**Authors:** Zhanjun Chen, Leyang Xiang, Longhai Li, Huohui Ou, Yinghao Fang, Yuyan Xu, Qin Liu, Zhigang Hu, Yu Huang, Xianghong Li, Dinghua Yang

**Affiliations:** 1 Unit of Hepatobiliary Surgery, Department of General Surgery, Nanfang Hospital, Southern Medical University, Guangzhou, 510515 Guangdong China; 2 Department of General Surgery, Affiliated Baoan Hospital of Shenzhen, Southern Medical University, Shenzhen, 518101 Guangdong China; 3 Department of Hepatobiliary Surgery, The First Affiliated Hospital of Jinan University, Guangzhou, 510632 Guangdong China; 4 Department of Hepatobiliary Surgery, Shunde Hospital, Southern Medical University (The First People’s Hospital of Shunde), Foshan, 528308 Guangdong China; 5 Department of Laboratory Medicine, Nanfang Hospital, Southern Medical University, Guangzhou, 510515 Guangdong China

**Keywords:** HCC, Linc00261, EMT, Stemness, TGF-β1, SMAD3

## Abstract

Emerging evidence suggests that long non-coding RNAs (lncRNAs) play important roles in the metastasis and recurrence of hepatocellular carcinoma (HCC). A kinds of lncRNAs were found to be involved in regulating epithelial–mesenchymal transition (EMT) or stem-like traits in human cancers, however, the molecular mechanism and signaling pathways targeting EMT and stemness remains largely unknown. Previously, we found that linc00261 was down-regulated in HCC and associated with multiple worse clinical pathological parameters and poor prognosis. Here, we show that linc00261 was down-regulated in TGF-β1 stimulated cells, and forced expression of linc00261 attenuated EMT and stem-like traits in HCC. Linc00261 also inhibited the tumor sphere forming in vitro and decreased the tumorigenicity in vivo. Furthermore, we revealed that linc00261 suppressed the expression and phosphorylation of SMAD3 (p-SMAD3), which could be core transcriptional modulator in TGF-β1 signaling mediated EMT and the acquisition of stemness traits. A negative correlation between linc00261 and *p-*SMAD3 was determined in HCC samples. Conclusion: Our study revealed that linc00261 suppressed EMT and stem-like traits in HCC cells by inhibiting TGF-β1/SMAD3 signaling.

**Supplementary Information:**

The online version contains supplementary material available at 10.1186/s12967-022-03276-z.

## Introduction

Hepatocellular carcinoma (HCC) is the sixth most common malignancy and fourth leading cause of cancer-associated death worldwide [1]. Liver transplantation, surgical resection, and local radiofrequency ablation are the main curative treatments for early staging HCC, however, the early recurrence and metastasis of HCC still make the post-operative survival unsatisfactory [2]; furthermore, the vast majority of newly diagnosed patients are always with intra- or extra-hepatic metastasis. Therefore, it is urgent to illuminating the underlying mechanisms of HCC metastasis.

It has been widely accepted that epithelial-mesenchymal transition (EMT) devoted to tumor metastasis and recurrence [3–5]. Epithelial cancer cells lose epithelial characteristics and gain the mesenchymal properties during EMT. EMT-related transcriptional factors (TFs), such as Snail/Slug, ZEB1/2, TWIST1, and signaling pathways, for instance, TGF-β1/SMAD, Wnt, VEGF, IGF, and Notch are crucial triggers and regulators for EMT [6]. Cancer stem cells (CSCs), a subgroup cancer cells with self-renewal and proliferative properties, were recently thought to be the seeds of tumor metastasis and recurrence [7]. By undergoing EMT, cancer cells could acquire the “stemness”, which establishes a close relationship between EMT, CSCs, and metastasis [8].

Liver CSCs have been reported to be enriched by several surface markers, including CD13, CD133, CD24, EpCAM, CD44 and CD90 [9–12]. However, the exact mechanism that liver CSCs maintain self-renewal characteristics has been rarely reported. Interestingly, pathways, such as TGF-β1 pathway, Wnt/β-catenin pathway, Notch pathway and Hedgehog pathway, and EMT-related TFs are increasingly shown to regulate the CSCs characteristics [13–15]. TGF-β1 signaling plays a dual role during the progression of HCC, it prevents the progression of HCC in the early stage, while promoting carcinogenesis in the late stage [16]. In HCC, TGF-β1 induced a partial EMT to maintain stemness characteristics, during which liver cancer cells acquire increased mobility and invasiveness [17]. However, the exact mechanism that TGF-β1 signaling regulates EMT and stemness needs further investigation.

Accumulating evidences indicated that long non-coding RNAs (lncRNAs) play a significant role in regulating EMT process in cancer cells [18]. Linc00261, also known as DEANR1, has been found dysregulated in numerous cancers, such as lung cancer [19], gastric cancer [20], endometrial cancer [21], and HCC [22]. Its downregulation could be associated with transcriptional inhibition by neighbor gene FOXA2, DNMT1-derived CpG islands methylation, and EZH2 catalyzed trimethylation of H3K27at lys27 (H3K27Me3) [23]. It inhibits cellular proliferation by promoting apoptosis, DNA damage, or G2/M cell cycle arrest, restrains cellular mobility and invasion by restricting the activation of Notch signaling [24] or accelerating the degradation of Slug [25]. Interestingly, as an endoderm differentiation specific lincRNA, linc00261 also specifically expressed in adult endoderm-derived tissues and liver shares the highest level; besides, our previous study and others revealed an inhibitory effect of linc00261 on EMT process and metastasis in HCC and gastric cancer. However, whether linc00261 deficiency modulated EMT induced acquisition of stemness, is still undefined.

In this study, we investigated the influence of linc00261 on regulating EMT and cancer stem cell-liked characteristics in HCC, and the exact role of linc00261 in modulating SMAD3, the key factor of TGF-β1 signaling. These findings may provide new strategies for the prevention and therapy for HCC metastasis.

## Materials and methods

### Cell lines

Liver cancer cell lines SMMC-7721 and Huh7 were bought from the Institutes of Biological Sciences, Chinese Academy of Sciences, Shanghai, China. HepG2 and Sk-hep-1 were purchased from American Type Culture Collection (ATCC; VA, USA), and MHCC-LM3 was obtained from Liver Cancer Research Institute, Zhongshan Hospital, Fudan University, Shanghai, China as a gift. Cell lines were cultured in DMEM (Gibco) with 10% fetal bovine serum (FBS; Gibco) at 37 °C in a humidified incubator with 5% CO_2_. Cells was treated with TGF-β1 (5 ng/ml) to induce the EMT.

### RNA isolation and quantitative real-time PCR (qRT-PCR)

Total RNA was isolated using Trizol reagent RNAisoPlus (Takara, Dalian, China) and reversely transcribed into cDNA using Primescript RT Master Mix (Takara), after which, expression of target gene was evaluated by qRT-PCR using SYBR Green Mix (Takara) according to the manufacturer′s instructions. The primes used were listed as follow: linc00261: 5′-GTCAGAAGGAAAGGCCGTGA-3′ (forward), 5′-TGAGCCGAGATGAACAGGTG-3′ (reverse); Nanog: 5′-TGAACCTCAGCTACAAACAG-3′ (forward), 5′-TGGTGGTAGGAAGAGTAAAG-3′ (reverse); SOX2: 5′-ACGCTCATGAAGAAGGATAAGT-3′ (forward), 5′-GAGCTGGTCATGGAGTTGTAC-3′ (reverse); OCT4: 5′-AGGTGGTCCGAGTGTGGTTC-3′ (forward), 5′-GAGGAGTACAGTGCAGTGAAGTG-3′ (reverse); Slug: 5′-CTGTGACAAGGAATATGTGAGC-3′ (forward), 5′-CTAATGTGTCCTTGAAGCAACC-3′ (reverse); Snail: 5′-CTTCCAGCAGCCCTACGAC-3′ (forward), 5′-CGGTGGGGTTGAGGATCT-3′ (reverse); ZEB1: 5′-AGCAGTGAAAGAGAAGGGAATGC-3′ (forward), 5′-GGTCCTCCTCAGGTGCCTCAG-3′ (reverse); 18SrRNA, 5′-GTAACCCGTTGAACCCCATT-3′ (forward), 5′-CCATCCAATCGGTAGTAGCG-3′ (reverse). 18S rRNA was used as internal control, and 2^−ΔΔCT^ method was applied to analyze expression of target genes.

### Small interfering RNA (siRNA) transfection and the construction of linc00261 overexpression cell lines

Liver cancer cell lines were seeded in the 6-well plates. Then the cells were washed three times with PBS and transfected with siRNA using lipofectamine 3000 (Invitrogen), and incubated for 48 h. The siRNA sequences for linc00261 were as follow: si-linc00261-1:5′-GAAAGCTGTAGCCATTCAA-3′, si-linc00261-2:5′-GCAATTAATTCAGGACACT-3′. The linc00261 overexpression lentivirus was constructed and bought from Genechem (Shanghai, China), and the construction of SMMC-7721-linc00261 overexpression model has been introduced in our previous research [23].

### Western blotting

RIPA lysis buffer (Beyotime, Shanghai, China) containing protein inhibitor, Phenylmethanesulfonyl fluoride (Beyotime), and the BCA kit (Beyotime) was used to determine the protein concentration after collecting the supernatant. The lysed proteins were separated on an SDS-PAGE gel and transferred to a PVDF membrane for immunoblotting analysis. The membranes were immersed in TBST solution containing 5% nonfat milk at room temperature for half an hour, incubated with the primary antibodies (Additional file 1: Table S2) overnight at 4 °C, and incubated with horse radish peroxidase-conjugated goat anti-rabbit secondary IgG antibody at room temperature for 1 h. Finally, the expression of proteins were detected using ECL substrate kit (Fdbio Science, Hangzhou, China) and FluorChem E system (ProteinSimple, CA, USA).

### Transwell migration and invasion assays

Cells were suspended in medium without FBS and a total of 1 × 10^5^cells were then added to the upper chambers, which were pre-coated with (for invasion assay) or without (for migration assay) Matrigel (BD Biosciences). Medium supplemented with 20% FBS was added to the lower chamber. Cells were cultured at 37 °C for another 48 h, after which, the migrated/invaded cells were fixed with 4% paraformaldehyde and stained using 0.5% crystal violet (Boster Biological Technology, Wuhan, China) at room temperature for 30 min. After washing with PBS, the chambers were air-dried and observed under an inverted light microscope (Olympus, Tokyo, Japan).

### Tumor-sphere culture

The tumor-sphere system mainly consisted of serum-free DMEM/F12 supplemented with 10 μl/ml B27 (Gibco)), 20 ng/ml of epidermal growth factor (EGF), 10 ng/ml of basic fibroblast growth factor (bFGF). Five hundred cell were seeded in a non-adherent 6-well plates (Corning) and maintained for 2 weeks. The non-adherent spheroid clusters (diameter ≥ 20 μm) [26] were observed under an inverted microscope (Olympus).

### Immunofluorescence staining

HCC cells seeded on coverslips were washed 3 times with PBS, fixed with 4% paraformaldehyde for 15 min, and permeabilized with 0.3% Triton X-100 at room temperature for 40 min for nuclear proteins. Then, the cells were blocked with 5% BSA for 30 min and stained with primary antibodies (Additional file 1: Table S2) overnight at 4 °C (Additional file 1: Table S1). After washing with PBS and incubation with Alexa fluor 594-conjugated goat-anti rabbit secondary antibody (Proteintech) at dark room for 1 h, the cells were incubated with 0.1% 4′,6-diamidino-2-phenylindole (DAPI) for 5 min, washed with PBS, and then observed under an inverted fluorescence confocal microscope (Olympus).

### Immunohistochemistry (IHC) analysis

After deparaffinization, the tissue sections (3 μm) were immersed in 10m Mcitrate buffer (pH 6.0) and subjected to microwave treatment for 15 min for antigen retrieval. The samples were subsequently immersed in 3% H_2_O_2_ for 30 min to block endogenous peroxidase, and then incubated with primary antibodies (Additional file 1: Table S2) at 4 °C overnight. The next day, the sections were incubated with horseradish peroxidase-conjugated goat-anti rabbit secondary antibody (ZSGB-BIO, Beijing, China), and developed with peroxidase substrate diaminobenzidine (DAB; ZSGB-BIO). Finally, the expression of proteins was observed and evaluated semi-quantitatively under an upright microscope (Olympus) as we previously reported.

### Patients specimens

HCC tissues and corresponding adjacent non-tumorous (NT) tissues were collected from Nanfang Hospital, Southern medical university between November 2010 and November 2016 in Nanfang Hospital. In paired HCC tissues, the relative linc00261 expression were analyzed by qRT-PCR, and IHC staining of E-cadherin, CD44, CD133, SMAD3 and p-SMAD3 was conducted.

### In vivo tumorigenicity

The animal experimental procedures were conducted strictly in accordance with the Guide for the Care and Use of Laboratory Animals. Male NSG mice (2-4 weeks) were bought from Biocytogen (BeiJing, China). A total of 5 × 10^6^ SMMC-7721 cells transfected with linc00261 overexpression or Vector lentivirus were subcutaneously implanted in the same mouse at different side. Tumor growth was recorded every 3 days, and the mice were sacrificed at the 18th day after injection.

### Statistical analysis

The statistical significance was determined by Student’s test (unpaired) or one-way ANOVA followed by a post hoc test when appropriate. Data were expressed as mean ± SD, and P value of 0.05 or less was considered significant. IBM SPSS 20.0 or GraphPad prism 5 software was used for the statistical analysis.

## Results

### Linc00261 is down-regulated in TGF-β1-induced EMT in HCC cell lines

**Fig. 1:**
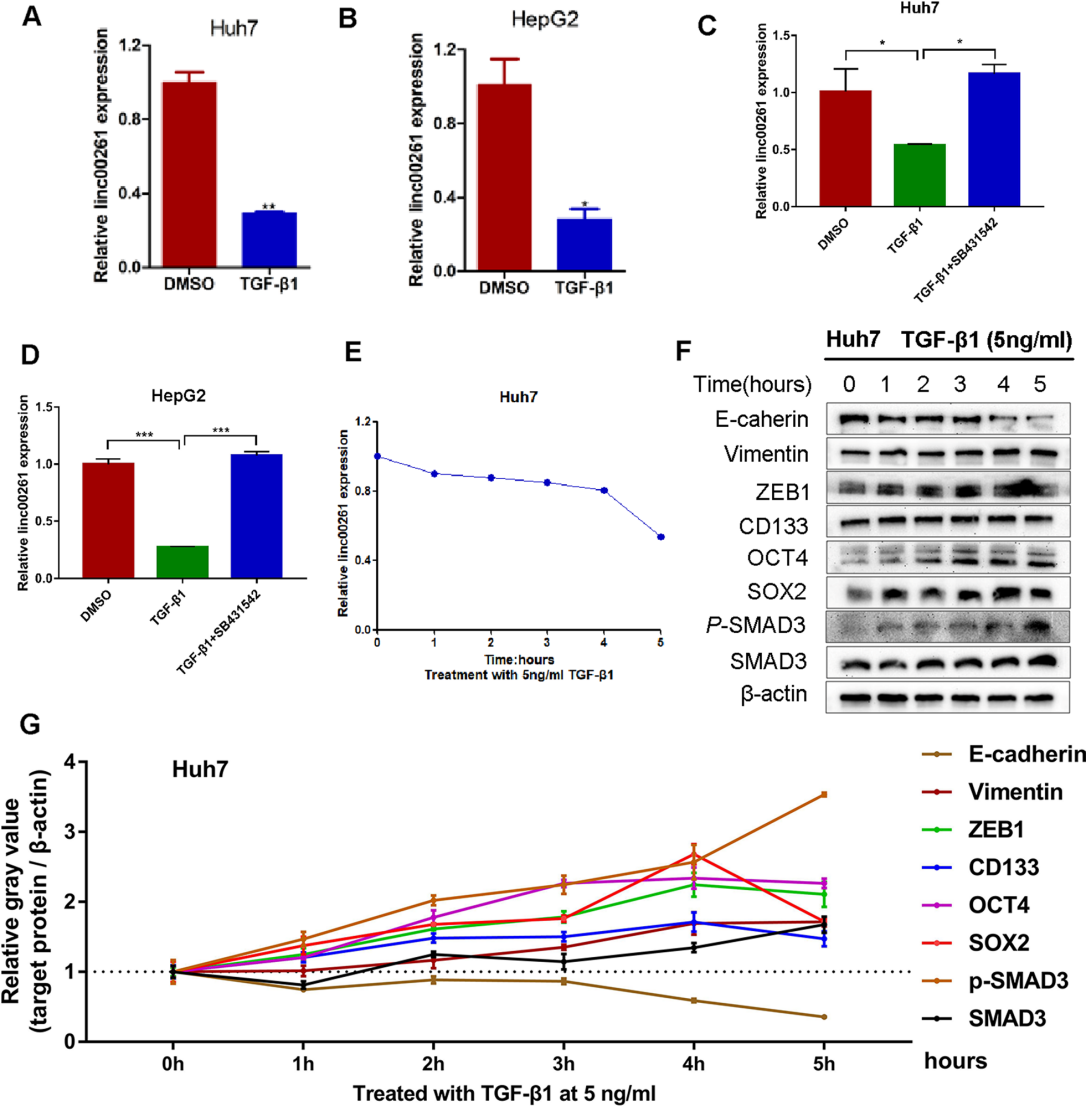
TGF-β1 induced the dysregulation of linc00261 and EMT/CSCs associated proteins in HCC cells. Linc00261 was down-regulated after TGF-β1 treatment in Huh7 (**A**) and HepG2 (**B**) cells determined by qRT-PCR. qRT-PCR analysis of linc00261 expression after treating with TGF-β1 and SB431542, a TGF-β1 inhibitor in Huh7 (**C**) and HepG2 (**D**) cell lines. **E** qRT-PCR analysis of linc00261 expression at time points of 0, 1, 2, 3, 4, and 5 h in Huh 7 cells after TGF-β1 treatment with 5 ng/ml. **F** Western blotting analysis of EMT, CSCs and TGF-β1 pathway related proteins after TGF-β1 treatment. **G** The relative gray value (normalized to β-actin) evaluations of the EMT, CSCs and TGF-β1 pathway related proteins in **E**.* *P* < 0.05; ** *P* < 0.01; *** *P* < 0.001

To explored the correlation between linc00261, TGF-β1 and TGF-β1-induced EMT and CSCs in HCC cells, TGF-β1 was treated for 48 h in two epithelial liver cancer cell lines (Huh7 and HepG2), transition from epithelial to fibroblast-like appearance was observed (Additional file 2: Fig. S1), and the linc00261 expression was significant down-regulated in Huh7 and HepG2 cells (Fig. 1A, B). When treated with SB431542, a specific TGF-β receptor (TGF-βR) inhibitor, TGF-β1-induced downregulation of linc00261 can be attenuated (Fig. 1C, D). Meanwhile, linc00261 was found down-regulated at the early time of TGF-β1 stimulation (Fig. 1E), with the expression of Vimentin, ZEB1 upregulated, and E-cadherin expression down regulated in Huh7 cells; besides, the expression of CSCs-relative markers, including CD133, OCT4, and SOX2 were increased which were reported to be associated with poor prognosis of HCC [27]. Besides, the expression of total and phosphorylated SMAD3 (p-SMAD3) were also increased after TGF-β1 stimulation (Fig. 1F, G). These results indicated that linc00261 is a TGF-β1-induced lncRNA and may be a target of TGF-β1 pathway.

### Linc00261 attenuated EMT and stem-like traits in HCC cells

**Fig. 2:**
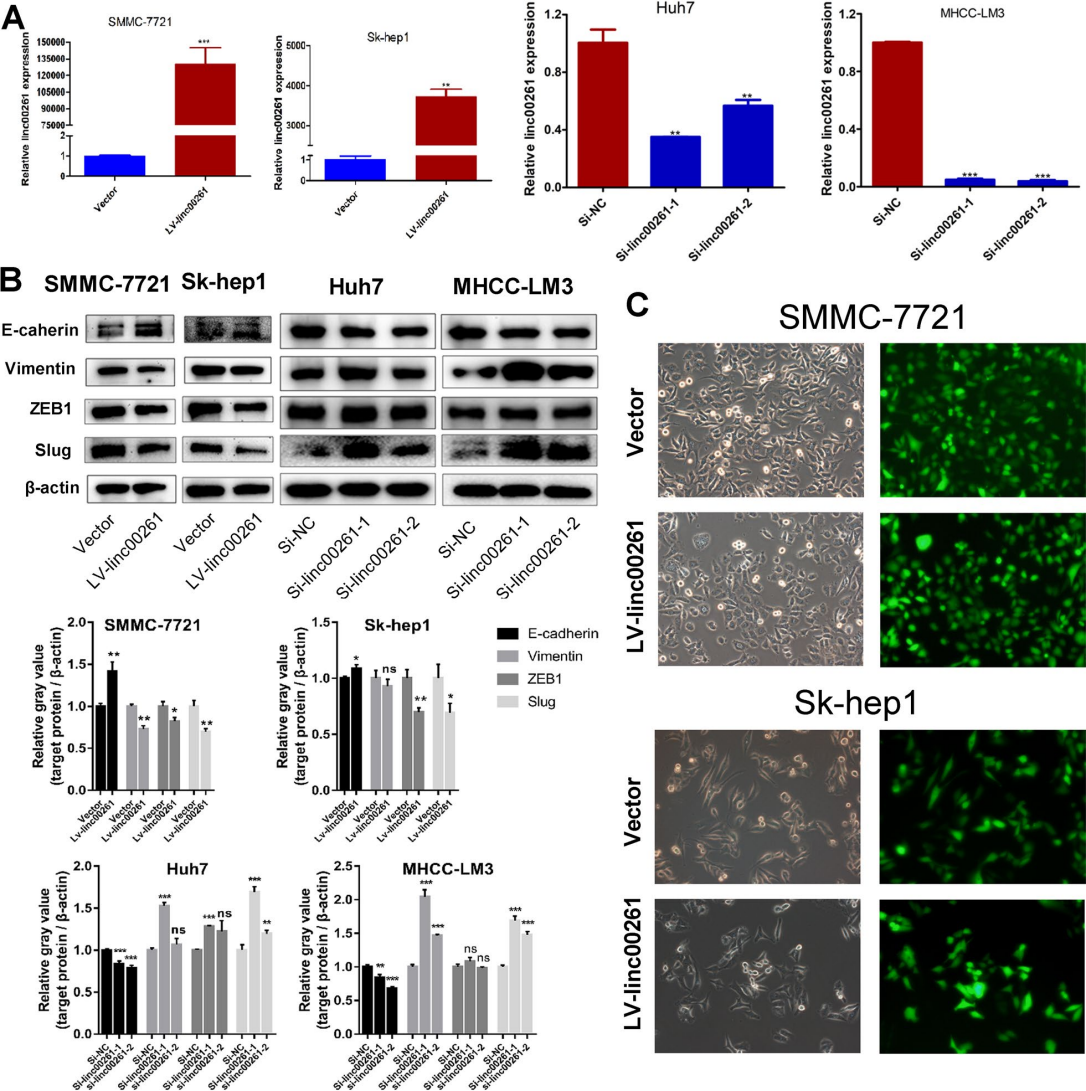
Linc00261 inhibits EMT in HCC cells. **A** Quantitative RT-PCR analysis of linc00261 expression in HCC cells after linc00261 overexpression (SMMC-7721, Sk-hep1) and linc00261 knockdown (Huh7, MHCC-LM3) using lentivirus and specific small interfering RNAs (siRNAs), respectively. **B** The protein expressions of epithelial (E-cadherin) and mesenchymal associated marker (Vimentin)/transcription factors (ZEB1 and Slug) were determined by western blotting after linc00261 overexpression in SMMC-7721 and Sk-hep1, or linc00261 knockdown in Huh7 and MHCC-LM3 cells. The gray value of these proteins were evaluated by normalizing to β-actin. **C **Linc00261 overexpression promotes the morphological transition from mesenchymal to epithelial states in SMMC-7721 and SK-Hep1. * *P* < 0.05; ** *P* < 0.01; *** *P* < 0.001

**Fig. 3:**
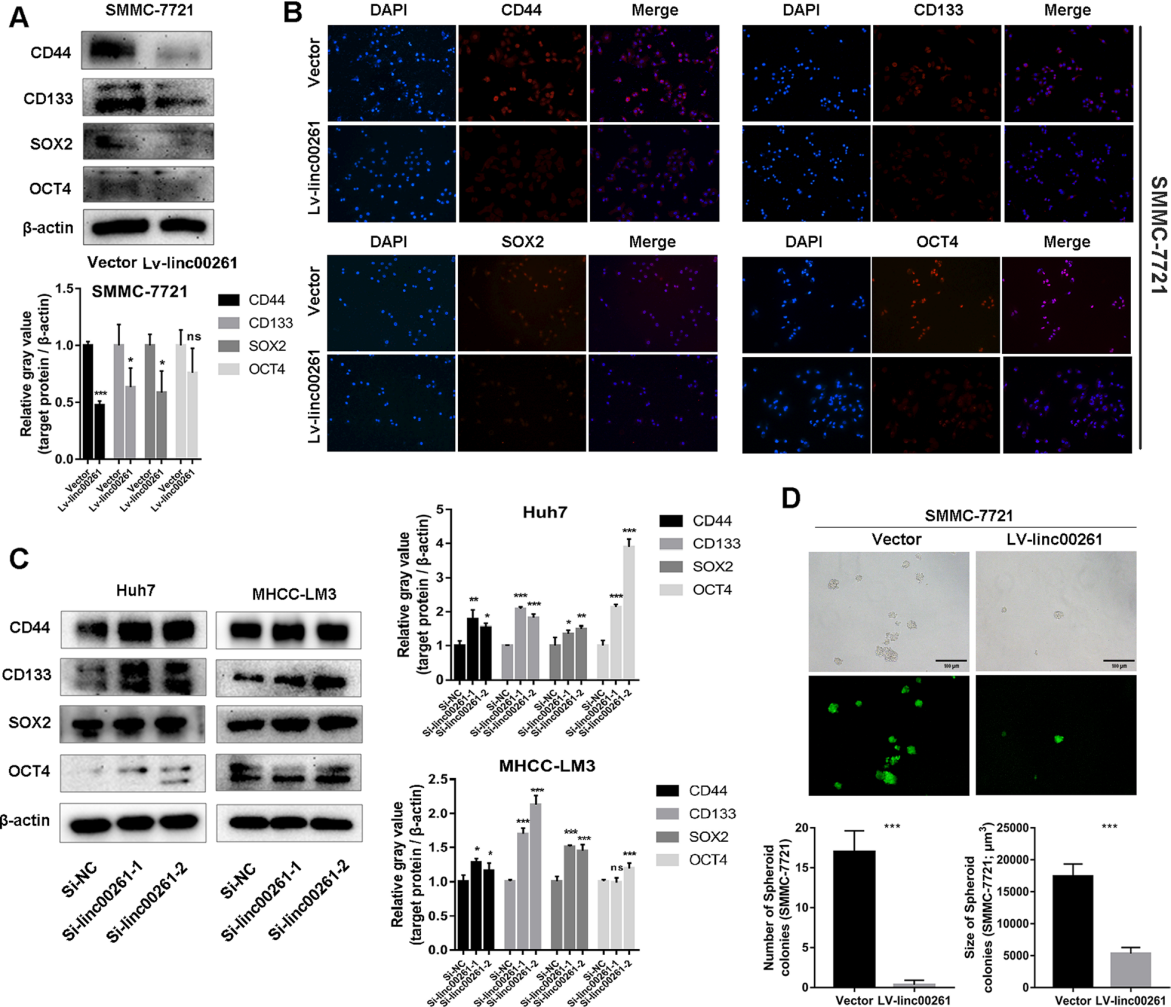
Linc00261 regulates CSCs related markers’ expression in HCC cells. **A**, **B** Western blotting analysis (**A**) and immunofluorescence staining analysis (**B**) of CSC-markers (CD44 and CD133) and -transcription factors (SOX2 and OCT4) in SMMC-7721 cells after overexpression of linc00261 using lentivirus. The histogram represents quantitative analyses of the relative gray values of the western blotting bands. **C **Western blotting analysis of CSC-markers (CD44 and CD133) and CSC-transcription factors (SOX2 and OCT4) protein levels in Huh7 cells and MHCC-LM3 cells after transfection with control siRNA or siRNAs against linc00261. The histogram represents quantitative analyses of the relative gray values of western blotting bands. **D** Linc00261 overexpression inhibits sphere formation both numbers and size in SMMC-7721 cells.* *P* < 0.05; ** *P* < 0.01; *** *P* < 0.001

To investigate the function of linc00261 in HCC, we constructed linc00261 stably overexpressionand transient knockdown models in 4 liver cancer cell lines (SMMC-7721 and Sk-hep1 for overexpression, Huh7 and MHCC-LM3 for transient knockdown; Fig. 2A). After linc00261 overexpression, the expression of epithelial maker, E-cadherin was upregulated, while the expression of mesenchymal maker (Vimentin) and EMT-associated TFs (ZEB1 and Slug) were decreased; and the transient knockdown models showed the opposite trends in Huh7 and MHCC-LM3 cell lines (Fig. 2B); moreover, the linc00261 overexpression cells acquired an epithelial-like appearance comparing to the control cells (Fig. 2C). Given that linc00261 could attenuate EMT in HCC, we next examined the influence of linc00261 on the stem-like traits in HCC cells. As expected, the western blotting analysis (Fig. 3A) and immunofluorescence staining (Fig. 3B) revealed that linc00261 overexpression inhibited the protein levels of CSCs markers (CD44 and CD133) and CSC-TFs (SOX2 and OCT4) in SMMC-7721. Conversely, the opposite changes of those proteins after linc00261 knockdown were observed in Huh7 and MHCC-LM3 cells (Fig. 3C). Further, linc00261 significantly inhibited the sphere formation in SMMC-7721 cells (Fig. 3D) and Sk-hep1 (Additional file 3: Fig. S2).

**Fig. 4:**
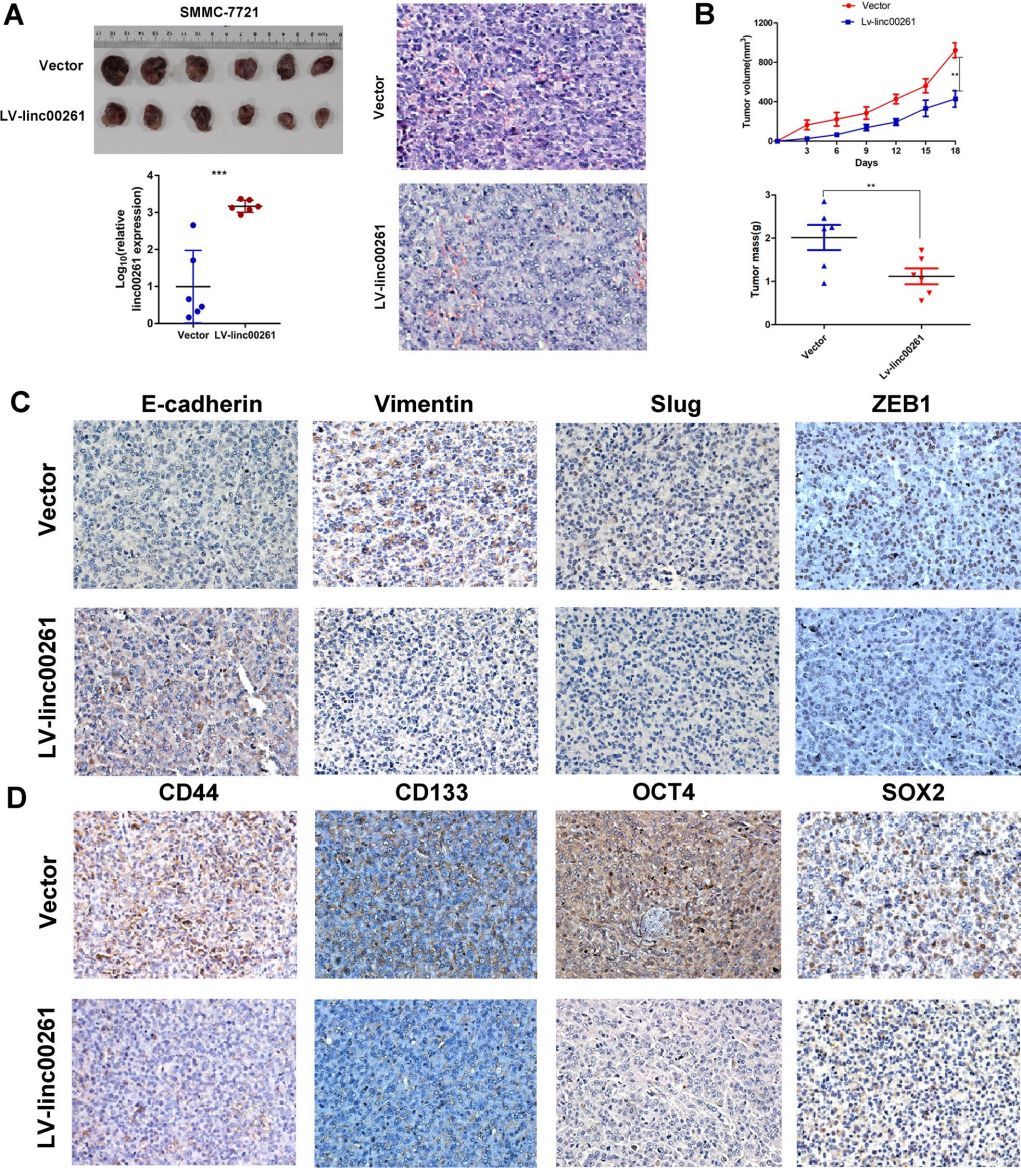
Linc00261 inhibits the growth of HCC cells in vivo. **A** Image of xenografts tissues after linc00261 overexpression in SMMC-7721 cells in vivo; the below histogram verified linc00261 expression in the xenograft tissues; the right pictures represent the HE staining of the xenograft tissues. **B** Linc00261 overexpression significant decrease the growth rates and tumor mass in SMMC-7721 cells in vivo. The (**C**, **D**) IHC analysis of EMT-related markers (**C**; E-cadherin, Vimentin, Slug, and ZEB1) and CSCs-related markers or transcription factors (**D**; CD44, CD133, OCT4, and SOX2) in xenograft tissues.* *P* < 0.05; ** *P* < 0.01; *** *P* < 0.001

In vivo, a subcutaneous xenograft tumor formation assay was conducted using SMMC-7721 linc00261 overexpression and vector cells (Fig. 4A). Overexpression of linc00261 significantly reduced the growth rates and tumor weights (Fig. 4B), and the IHC staining in xenograft tumors indicated that both the changes of expression of EMT-related (E-cadherin, Vimentin, Slug and ZEB1) and CSCs-related proteins (CD44, CD133, OCT4 and SOX2) were in accordance with in vitro assays (Fig. 4D, E). Taken these together, our findings strongly suggest that linc00261 attenuates EMT and is associated with stem-like traits in HCC cells.

### Linc00261 reverses TGF-β1 induced EMT and inhibits TGF-β1-stimulated target genes’ expression

**Fig. 5:**
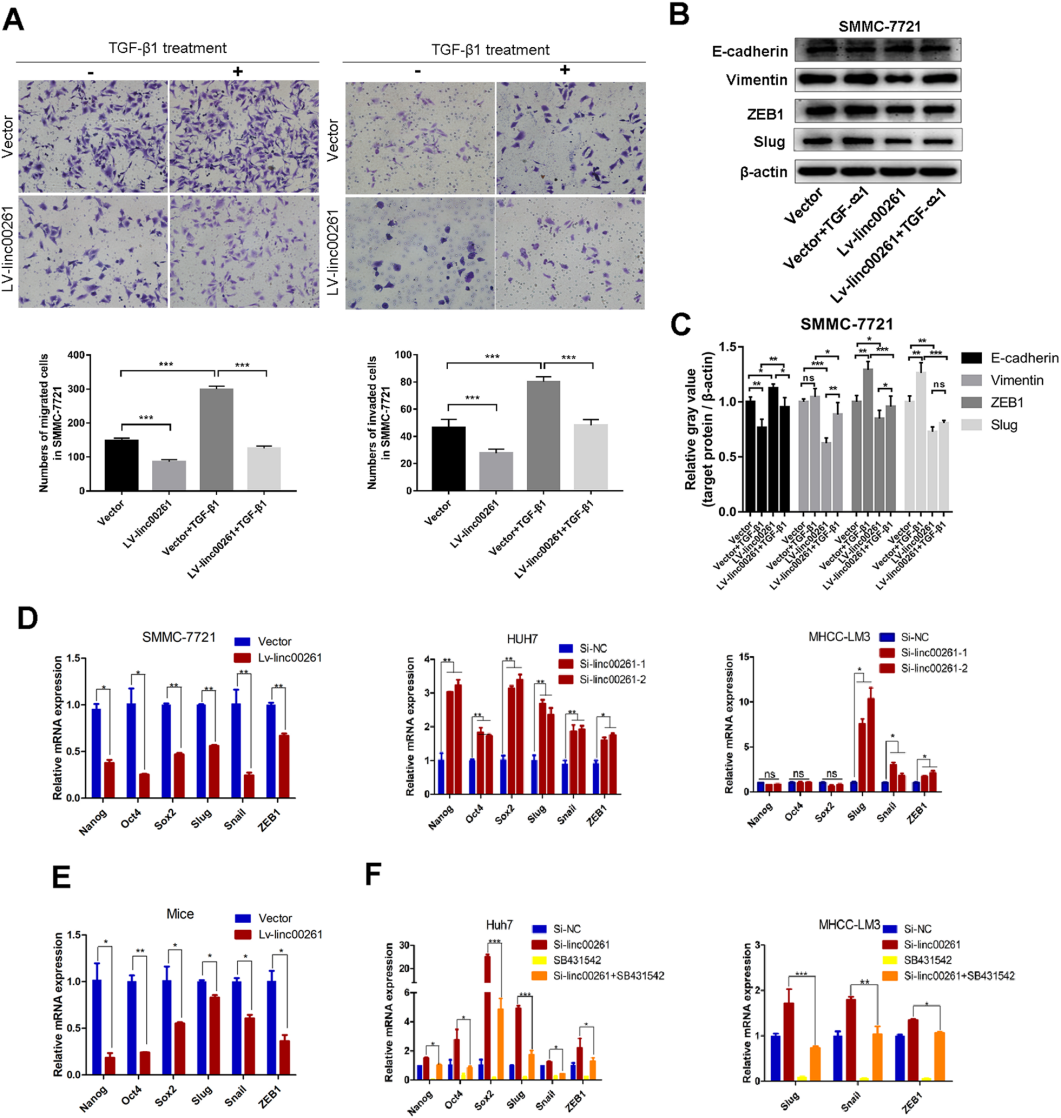
Linc00261 reverses TGF-β1 induced EMT and inhibits TGF-β1-stimulated target genes’ expression. **A **Transwell migration and invasion assays in linc00261 overexpression or control cells with or without TGF-β1 (5 ng/ml) treatment in SMMC-7721 cells. Western blotting analysis of EMT-related protein in vector or linc00261 overexpression SMMC-7721 cells with or without TGF-β1 treatment at 5 ng/ml (**B**); The histogram represents the gray values evaluation of these western blotting bands using image J software (**C**). **D** Linc00261 overexpression inhibits, while linc00261 down-regulation upregulates EMT-TFs (Slug, Snail and ZEB1) and CSC-TFs (Nanog, OCT4 and SOX2) at the RNA levels determined by qRT-PCR. **E** Linc00261 overexpression inhibits the EMT-TFs (Slug, Snail and ZEB1) and CSC-TFs (Nanog, OCT4 and SOX2) at RNA levels in vivo. **F** SB431542 inhibits the up-regulation of EMT-TFs (Slug, Snail and ZEB1) and CSC-TFs (Nanog, OCT4 and SOX2) induced by linc00261 knockdown in Huh7 and MHCC-LM3 cells.*P < 0.05; ** *P* < 0.01; *** *P* < 0.001

To determine whether linc00261 regulate cellular migration, invasion and EMT by influencing TGF-β1 pathway in HCC cells, the LV-linc00261 and vector SMMC-7721 cells were treated with TGF-β1. Overexpression of linc00261 abolished the migration and invasion-promoting effects induced by TGF-β1 (Fig. 5A), and remained the cells at an epithelial-like appearance (Additional file 4: Fig. S3). The western blots also showed that TGF-β1 significantly elevated the expression of ZEB1 and Slug, however, linc00261 attenuated TGF-β1 induced upregulation of them; and the changes of E-cadherin was in opposite direction with ZEB1 and Slug (Fig. 5B, C).

To study the role of linc00261 in TGF-β1 pathway, we further investigated whether linc00261 had an influence on the downstream targets of TGF-β1 pathway, including the key TFs of CSCs (NANOG, OCT4 and SOX2) and EMT (Snail, Slug and ZEB1). Surprisingly, overexpression of linc00261 significantly inhibited the mRNA expression of the downstream genes of TGF-β1 signaling in SMCC-7721 cell line (Fig. 5D), and knockdown of linc00261 activated them in Huh7 and MHCC-LM3 (partially) cell lines (Fig. 5D). The changes of these TFs in mRNA levels in xenograft tumors were in accordance with those in vitro SMMC-7721 cell line (Fig. 5E). In addition, knockdown of linc00261 significantly upregulate the downstream genes in mRNA levels of TGF-β1 pathway in Huh7 and MHCC-LM3 (only Slug, Snail, and ZEB1) cells, but rapidly, the activated TGF-β1 signaling was blocked by TGF-βR inhibitor, SB431542 (Fig. 5F), which indicated that linc00261 has a suppressive role in TGF-β1 pathway.

### Linc00261 blocks TGF-β1signaling via inhibiting SMAD3 expression and phosphorylation

**Fig. 6:**
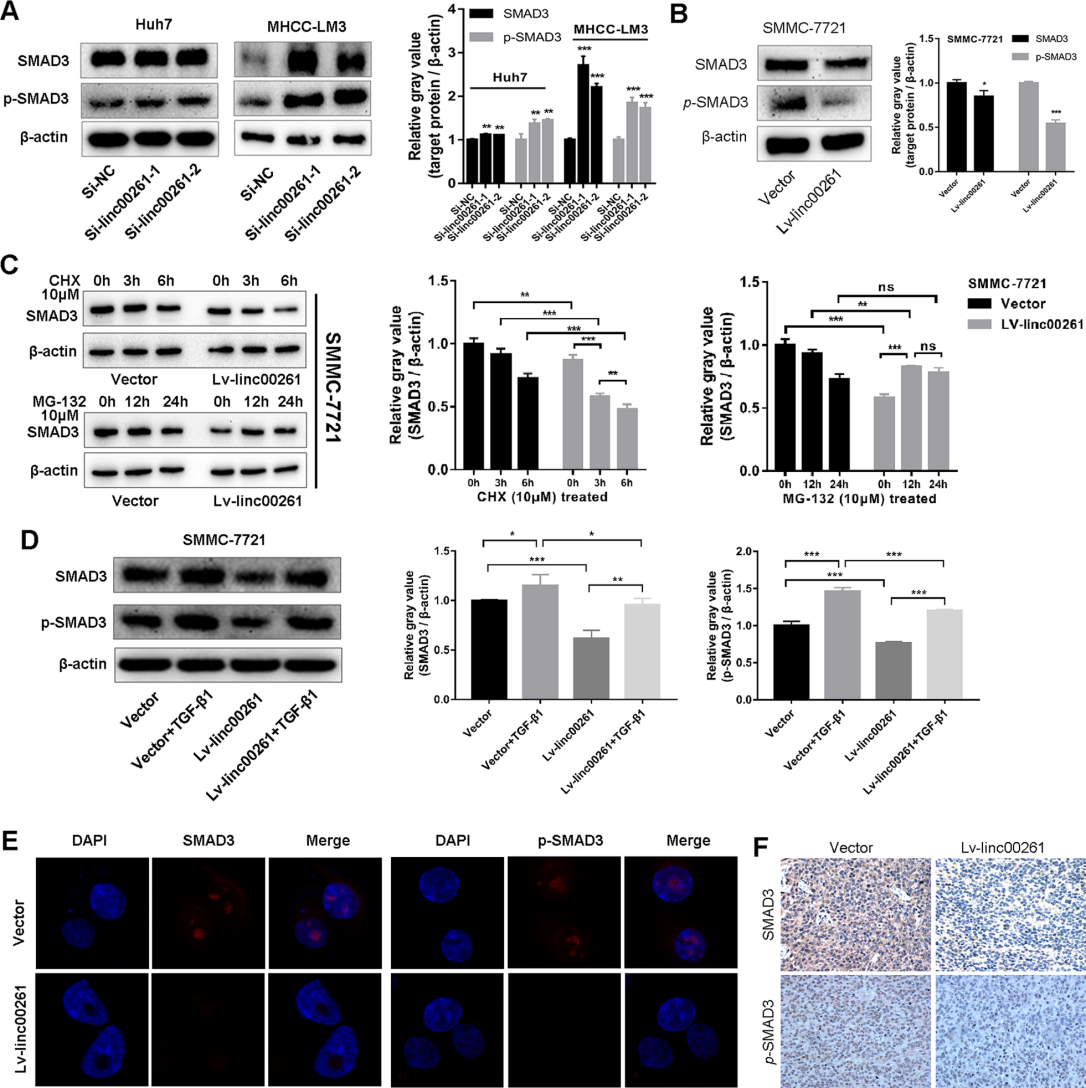
Linc00261 blocks TGF-β1 signaling via inhibiting SMAD3 expression and phosphorylation. Western blotting analysis of SMAD3 and *p-*SMAD3 protein after linc00261 knockdown (**A**; Huh7 and MHCC-LM3) or linc00261 overexpression (**B**; SMMC-7721). **C** Linc00261 overexpression promotes the ubiquitination dependent degradation of SMAD3 reflected by CHX (10 μM) and MG132 (10 μM) treatment in SMMC-7721 cells. **D** Western blotting analysis of total or p-SMAD3 protein expression in linc00261 overexpression or control cells treated with or without TGF-β1 in SMMC-7721 cells. **E** Immunofluorescence staining of SMAD3 and *p-*SMAD3 protein after linc00261 overexpression in SMMC-7721 cells. **F** IHC analysis of SMAD3 and *p-*SMAD3 protein levels in vivo. The histograms represent the evaluation of gray values of these western blotting bands. * *P* < 0.05; ** *P* < 0.01; *** *P* < 0.001

Our previous study revealed a direct combination of linc00261 with SMAD3 protein [23], to explore the exact interaction between lin00261 and SMAD3, we measured the total and phosphorylated (ser423/425) SMAD3 in HCC cells after knocking down or over-expressing linc00261. The total protein level of SMAD3, and p-SMAD3 (ser423/425) were reduced in linc00261 overexpression cells, whereas increased in linc00261 knocked-down cells (Fig. 6A, B). After treatment with CHX (10 μM) for 0, 3, and 6 h, the SMAD3 protein was observed decreasing much more rapidly in Lv-linc00261 cells comparing to vector cells, however, the SMAD3 protein was increasing after treatment with MG-132 (10 μM) for 0, 12, and 24 h in Lv-linc00261 cells, but still significantly lower than vector group (Fig. 6C), which indicated that linc00261 facilitates SMAD3 degradation by ubiquitin–proteasome pathway. Moreover, the western blotting and immunofluorescence staining demonstrated that linc00261 reduce TGF-β1-induced upregulation of total SMAD3 and p-SMAD3 (Fig. 6D, E); and IHC staining using the xenograft tumors further revealed that the SMAD3 protein in both cytoplasm and nucleus, and the p-SMAD3, especially in nucleus were obviously decreased in Lv-linc00261 groups (Fig. 6F). These results indicated that linc00261 suppresses both SMAD3 and p-SMAD3 expression partially through ubiquitin–proteasome pathway.

### Clinical relation of linc00261 with *p*-SMAD3 expression in human HCC tissues

**Fig. 7:**
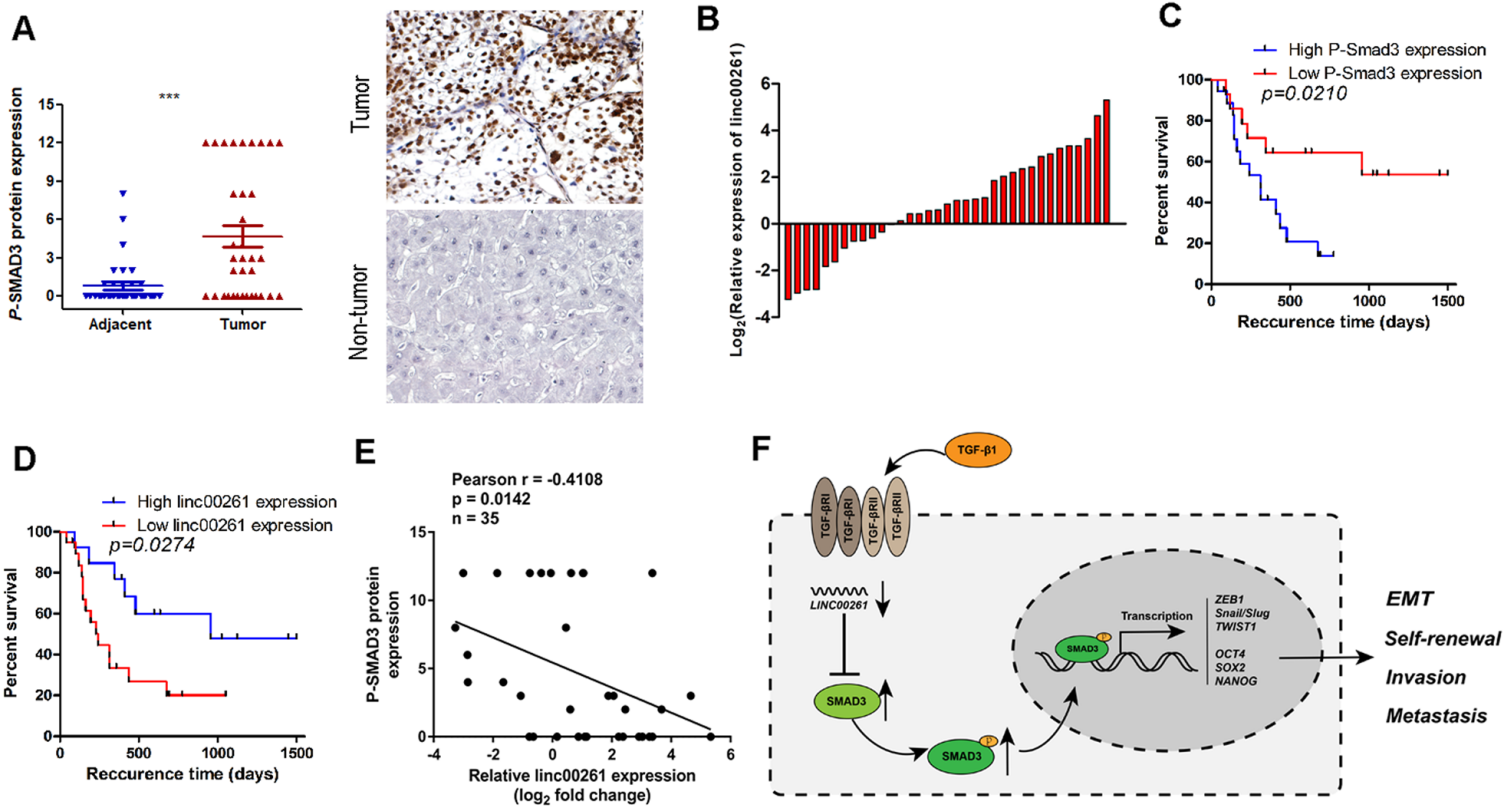
Correlation between linc00261 and *p*-Smad3 in patient samples.** A** IHC analysis of *p*-SMAD3 protein expression in 35 human HCC tissues and paired normal tissues. **B** qRT-PCR analysis of relative linc00261 expression in the same 35 human HCC tissues and paired normal tissues. **C** High *p*-SMAD3 protein expression was associated with poor prognosis in human HCC patients. **D** Low linc00261 expression was associated with poor prognosis in human HCC patients. **E** Pearson correlation of linc00261 with p-SMAD3 protein in 35 human HCC tissues. **F **Schematic diagram of linc00261 induced inhibition of EMT and stemness in HCC cells. *** *P* < 0.001

To further validate the correlation between linc00261 and *p*-SMAD3 (Ser423/425) expression, we tested the expressionof *p*-SMAD3 by immunohistochemical (IHC) analysis and linc00261 expression by qRT-PCR in tissues from the same cohort of HCC patients (n = 35; Fig. 7A, B. The *p*-SMAD3 was expressed at high levels in tumor tissues compared to non-tumor regions (Fig. 7A). Survival analysis showed that higher *p*-SMAD3 expression or lower linc00261 expression predict poor recurrence-free survival (RFS) in HCC patients (Fig. 7C, D). Further, there was a negative correlation between linc00261 and *p*-SMAD3 protein levels (Fig. 7E). Taken together, our results indicated that linc00261 attenuated EMT and stem-like traits by facilitating SMAD3 degradation and inhibiting SMAD3 phosphorylationin HCC.

## Discussion

TGF-β1 had been reported to be a key factor associated with tumor EMT and stemness, leading to tumor metastasis [26, 28]. In this study, we identified that linc00261 was down-regulated after TGF-β1 treatment, and linc00261 attenuated EMT and stem-like traits in liver cancer cells. Mechanistically, linc00261 facilitates SMAD3 degradation through ubiquitin–proteasome pathway and SMAD3 phosphorylation, thereby inhibiting HCC metastasis.

It’s well known that LncRNAs function as tumor suppressors or promoters through regulating EMT and CSCs by targeting multiple signaling pathways, including TGF-β1 pathway [29]. The function of linc00261 has been investigated in multiple cancers, it suppressed lung and gastric cancer progression and metastasis by attenuated EMT [25, 30], and function as a tumor suppressor in varies of human cancers by sponging with miRNA or affecting pathways [31–33]. In contrast, Gao et al. found that linc00261 was at high expression in cholangiocarcinoma, and its higher expression predicted a poorer prognosis [34]. Our preview study had demonstrated that patients with low expression of linc00261 had a poor progression in HCC, and cells after linc00261 knockdown had increased migratory and invasive capabilities [22]; moreover, our another study revealed that linc00261 suppresses the formation of microvascular invasion, EMT, and metastasis of HCC through transcriptional upregulation of FOXA2 by recruiting SMAD3 to the FOXA2 promotor regions [23]. LncRNAs can act as cis or trans to regulate genes expression in a precise temporal and spatial manners [35]. Considering the close relation of linc00261 with SMAD3, and the observed effect of linc00261 on EMT and CSCs traits, we further investigated whether linc00261 was involved in TGF-β1-regulated progression of HCC.

Interestingly, we observed that linc00261 was significantly down-regulated after treatment with TGF-β1, which is consistent with TGF-β1 induced-suppression of linc00261/Foxa2 in lung cancer cells [19]. According to our findings, overexpression of linc00261 induced an epithelial-like appearance, inhibited the tumor spheres formation, and also abolished TGF-β1-induced EMT, migration, and invasion in SMMC-7721; moreover, both linc00261 knockdown and overexpression affect the mRNA and protein expressions of the EMT-TFs (Slug and ZEB1) and CSCs-TFs (OCT4 and SOX2), the core downstream targets of TGF-β1 pathway, besides, the activated TGF-β1 signaling after linc00261 knockdown was blocked by TGF-βR inhibitor, SB431542. All these results demonstrated that linc00261 down-regulation is necessary for TGF-β1-induced EMT, and even CSCs traits acquisition. However, the exact mechanism that TGF-β1 suppresses linc00261 expression, and even TGF-β1 could forms a feedback loop with linc00261, still need further investigation.

TGF-β1/Smad signaling has a dual role among the tumor igenicity depending on cellular context and tumor stages [36, 37]. After TGF-β signaling activation, SMAD2 and SMAD3 acquire phosphorylation and then translocate into nucleus, thereafter combined with SMAD4 to trans activate downstream target genes [38, 39]. The functions of SMAD3 in HCC were still controversial; some reports showed that it was a tumor suppressor, while others proposed that it was a tumor promoter. A recent study had demonstrated that SMAD3 could promote migration, invasion, and metastasis of HCC cells in vitro and in vivo, binding directly to PTPRε promoters to activate its expression, and then feedback to activate TGF-β1/SMAD3 signaling to promote HCC metastasis [40]. However, our preview study indicated that linc00261 guides SMAD3 protein to the promoter region of FOXA2 genome to enhance its transcription, thereafter contributes to the prevention of HCC progression [23]. Herein, we found that linc00261 decreases TGF-β1-induced upregulation of SMAD3 and *p*-SMAD3; furthermore, linc00261 promotes the degradation of SMAD3 by ubiquitin–proteasome pathway and inhibits the phosphorylation of SMAD3 in vitro and in vivo. These results indicated that linc00261 inhibits TGF-β/SMAD3 signaling to prevent the progression of HCC, which could be a strong evidence supporting the oncogenic role of SMAD3.

The intracellular regulators of TGF-β1 signaling includes Smad-dependent and -independent pathways, the former is known as receptor-regulated SMADs (SMAD1, 2, 3, 5, and 8), inhibitory SMADs (SMAD 6 and 7), and common mediator SMADs. The inhibitory SMADs antagonize the receptor-regulated SMADs’ activity by interacting with TGF-βR1, and then prevent the R-SMADs from phosphorylation, followed with degradation via the ubiquitin proteasome pathway [41]. Therefore, linc00261 could interact with inhibitory SMADs, or potentially E3 ubiquitin ligase and phosphorylases to suppress SMAD3 phosphorylation and accelerate ubiquitin proteasome pathway-based degradation. Besides, our previous study demonstrated a lower expression of linc00261 in cytoplasm compared to nucleus in liver cancer cell lines [23], which supports the current phenomenon that reduced linc00261, especially cytoplasmic linc00261, allows SMAD3 to get phosphorylation (Ser423/425), and escape from ubiquitination and degradation, thereby translocate into the nucleus, and ultimately promote the progression of HCC.

In conclusion, our results demonstrated that TGF-β1-induced deficiency of linc00261 facilitates EMT and stemness via inhibiting SMAD3 in HCC. It is the first study to reveal the inhibitory role of linc00261 on TGF-β1/SMAD3 signaling, providing a novel mechanism underlying TGF-β1-induced EMT and stem-like traits in HCC. Also, our work provides a new potential therapeutic target for the treatment of HCC.

## Supplementary Information

**Additional file 1: Table S1.** Inhibitors and stimulant used in this research. **Table S2.** Primary antibodies and its dilutions used for western blotting, immunohistochemical, immunofluorescence staining.

**Additional file 2: Figure S1.** TGF-β1 treatment for 48 h at a concentration of 5 ng/ml promotes the morphological transition from epithelial to mesenchymal states in HepG2, Huh7, and SNU-449 cells. The right histogram indicated linc00261 expression determination using qRT-PCR after TGF-β1 stimulation.

**Additional file 3: Figure S2.** Linc00261 overexpression inhibits sphere formation both in numbers and size in Sk-hep1 cells. **P < 0.01; *** *P* < 0.001.

**Additional file 4: Figure S3.** TGF-β1 treatment for 48 hours at a concentration of 5ng/ml promotes the morphological transition from epithelial to mesenchymal states in SMMC-7721 vector cells, linc00261 overexpression reverses this transition.
