# Supplementary material for: TGF-β1 induced deficiency of linc00261 promotes epithelial–mesenchymal-transition and stemness of hepatocellular carcinoma via modulating SMAD3
Source: J Transl Med. 2022 Feb 5;20:75. doi: 10.1186/s12967-022-03276-z (PMC8818189; doi:10.1186/s12967-022-03276-z)
Supplement: Supplementary file 1 — Additional file 1: Table S1. Inhibitors and stimulant used in this research. Table S2. Primary antibodies and its dilutions used for western blotting, immunohistochemical, immunofluorescence staining. [file 12967_2022_3276_MOESM1_ESM.docx]

Additional file

Table S1 Inhibitors and stimulant used in this research

| **Inhibitors/stimulant** | **concentrations** | **Incorporation** | **Catalog number** |
| --- | --- | --- | --- |
| SB431542 | 10mM | Selleck | S1067 |
| CHX | 10μM | MCE | HY-12320 |
| MG-132 | 10μM | MCE | HY-13259 |
| TGF-β1 | 5ng/mL | PeproTech | AF-100-21C-100 |
| DMSO | - | MP bio | 67-68-5 |

| **Primary antibodies** | **WB** | **IHC-P** | **IF** | **Specificity** | **catalog**  **number** | **Incorporation** |
| --- | --- | --- | --- | --- | --- | --- |
| E-cadherin | 1:1000 | 1:300 |  | Rabbit monoclonal | 3195 | CST |
| Vimentin | 1:1000 | 1:200 |  | Rabbit monoclonal | 5741 | CST |
| ZEB1 | 1:1000 | 1:300 |  | Rabbit monoclonal | 70512 | CST |
| Slug | 1:1000 |  | 1:50 | Rabbit monoclonal | 9585 | CST |
| CD133 | 1:1000 | - | - | Rabbit monoclonal | 64326 | CST |
| SOX2 | 1:1000 | 1:300 | 1:50 | Rabbit polyclonal | 14962 | CST |
| SMAD3 | 1:1000 | 1:100 | - | Rabbit polyclonal | ab28379 | Abcam |
| *p*-SMAD3 (Ser423/425) | 1:1000 | 1:50 | 1:50 | Rabbit monoclonal | 9520 | CST |
| OCT4 | 1:1000 | 1:200 | 1:200 | Rabbit monoclonal | 2750 | CST |
| ACTB | 1:1000 | - | - | Rabbit polyclonal | 20536-1-AP | Proteintech |

Table S2 Primary antibodies and its dilutions used for western blotting, immunohistochemical, immunofluorescence staining
