# Supplementary figures and images for: TGF-β1 induced deficiency of linc00261 promotes epithelial–mesenchymal-transition and stemness of hepatocellular carcinoma via modulating SMAD3
Source: J Transl Med. 2022 Feb 5;20:75. doi: 10.1186/s12967-022-03276-z (PMC8818189; doi:10.1186/s12967-022-03276-z)

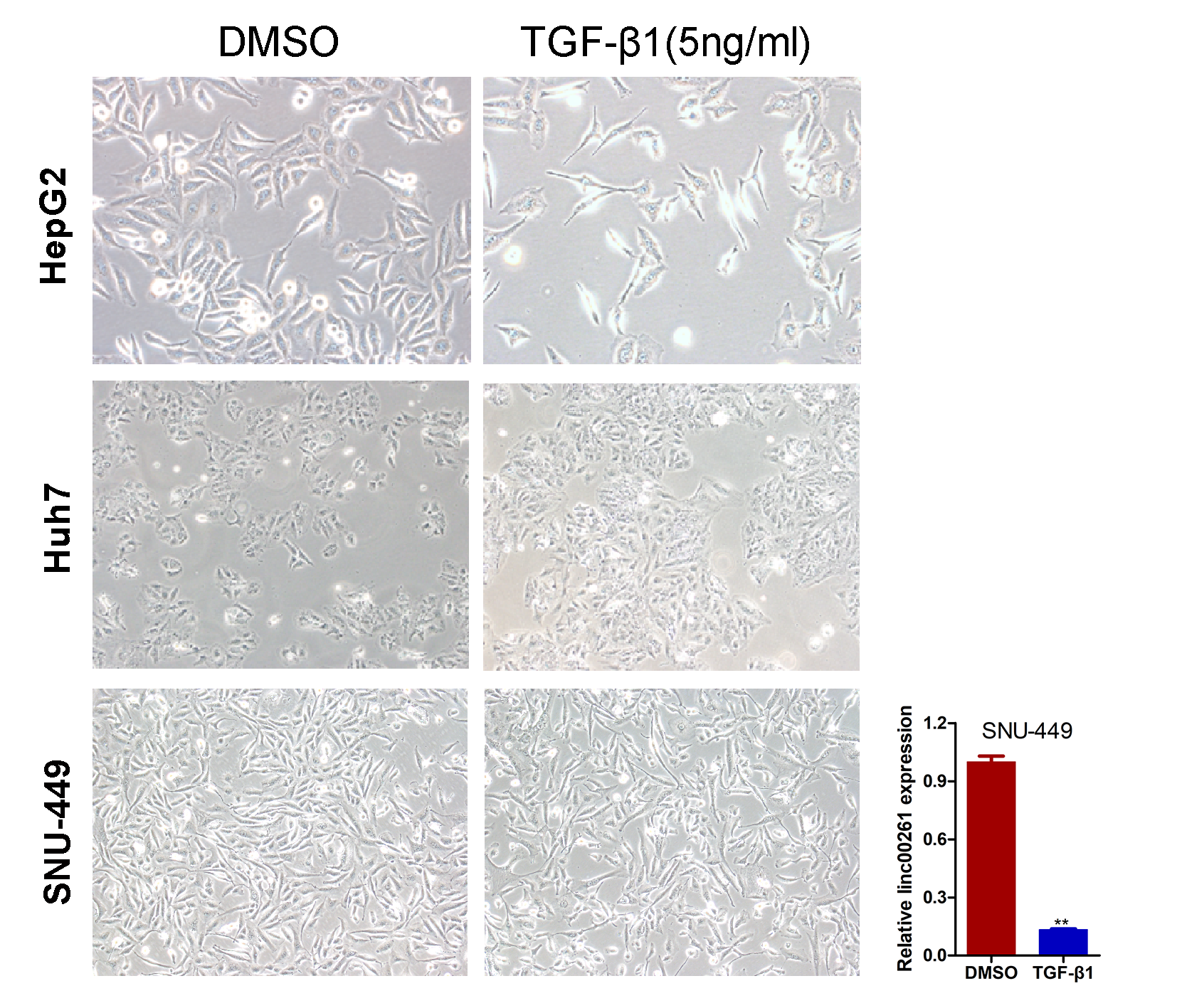

Supplement: Supplementary file 2 — Additional file 2: Figure S1. TGF-β1 treatment for 48 h at a concentration of 5 ng/ml promotes the morphological transition from epithelial to mesenchymal states in HepG2, Huh7, and SNU-449 cells. The right histogram indicated linc00261 expression determination using qRT-PCR after TGF-β1 stimulation. [file 12967_2022_3276_MOESM2_ESM.tif]

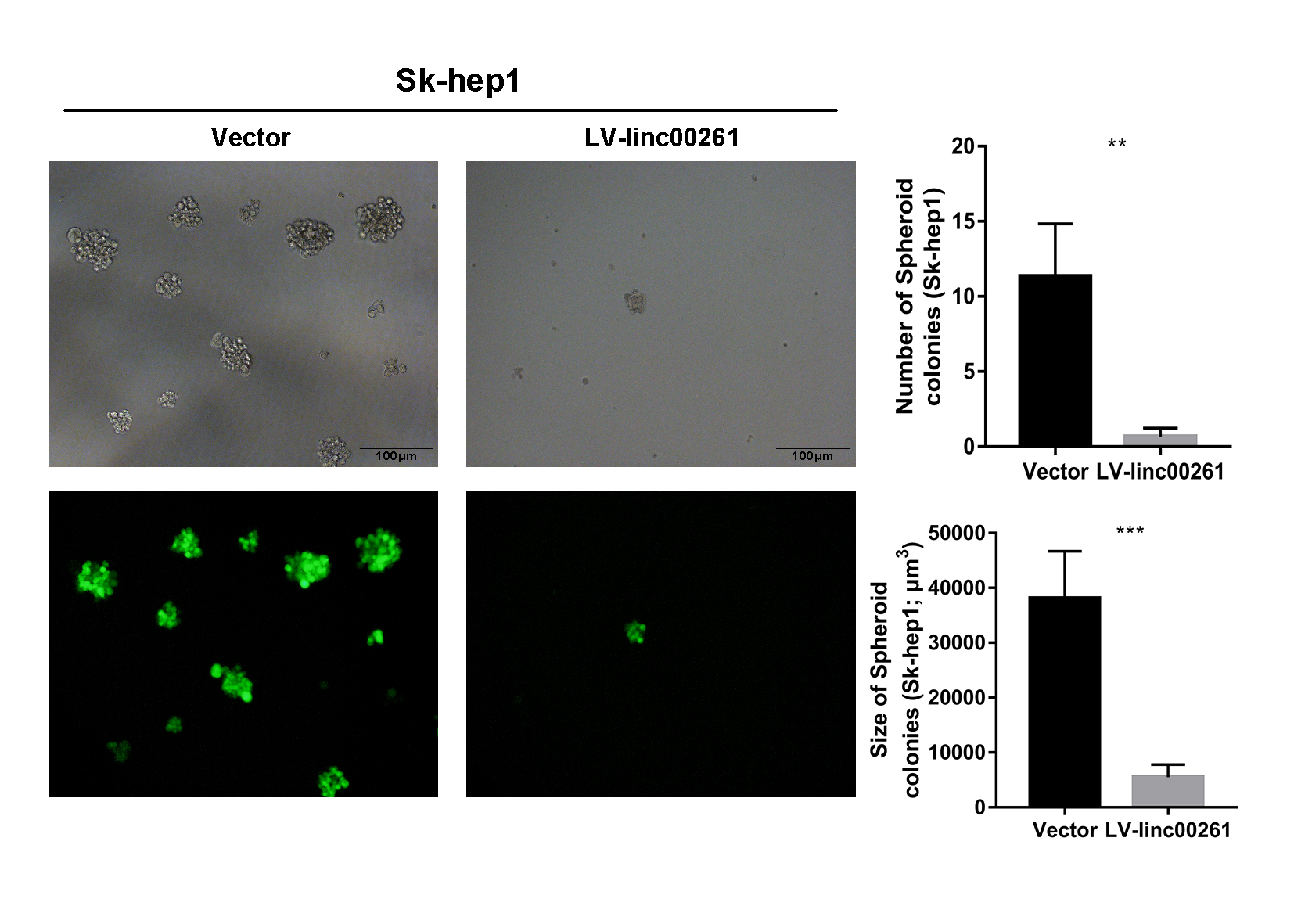

Supplement: Supplementary file 3 — Additional file 3: Figure S2. Linc00261 overexpression inhibits sphere formation both in numbers and size in Sk-hep1 cells. **P < 0.01; *** P < 0.001. [file 12967_2022_3276_MOESM3_ESM.tif]

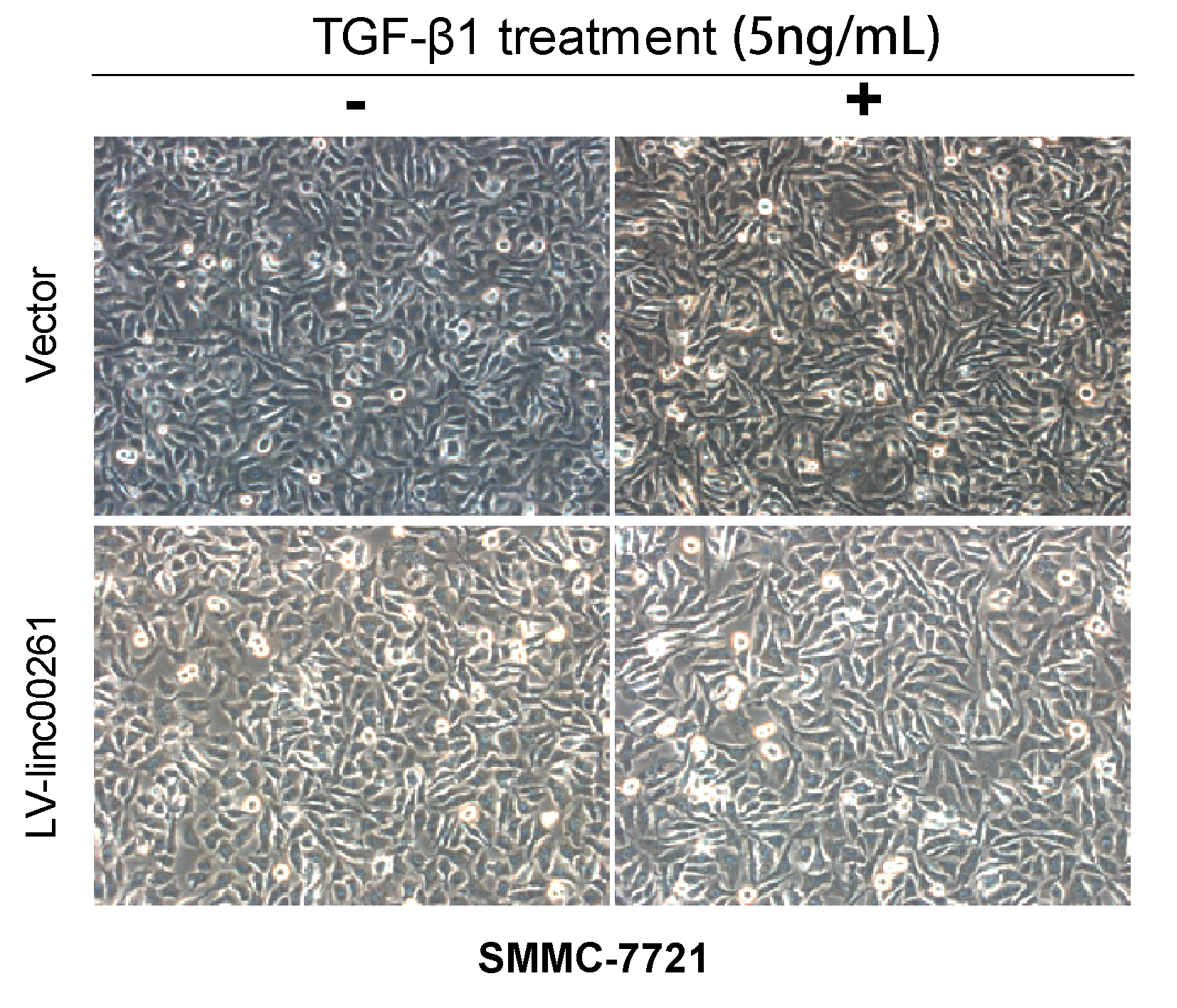

Supplement: Supplementary file 4 — Additional file 4: Figure S3. TGF-β1 treatment for 48 hours at a concentration of 5ng/ml promotes the morphological transition from epithelial to mesenchymal states in SMMC-7721 vector cells, linc00261 overexpression reverses this transition. [file 12967_2022_3276_MOESM4_ESM.tif]
